# Growth, structure and stability of sputter-deposited MoS_2_ thin films

**DOI:** 10.3762/bjnano.8.113

**Published:** 2017-05-22

**Authors:** Reinhard Kaindl, Bernhard C Bayer, Roland Resel, Thomas Müller, Viera Skakalova, Gerlinde Habler, Rainer Abart, Alexey S Cherevan, Dominik Eder, Maxime Blatter, Fabian Fischer, Jannik C Meyer, Dmitry K Polyushkin, Wolfgang Waldhauser

**Affiliations:** 1 JOANNEUM RESEARCH - MATERIALS, Institute for Surface Technologies and Photonics, Leobner Straße 94, A-8712 Niklasdorf, Austria; 2 Faculty of Physics, University of Vienna, Boltzmanngasse 5, A-1090 Vienna, Austria; 3 Institute of Solid State Physics, Graz University of Technology, Petersgasse 16, A-8010 Graz, Austria; 4 Photonics Institute, Vienna University of Technology, Gusshausstraße 27–29, A-1040 Vienna, Austria; 5 Danubia NanoTech, Ilkovicova 3, SVK-84104, Bratislava, Slovakia; 6 Department of Lithospheric Research, University of Vienna, Althanstraße 14, A-1090 Vienna, Austria; 7 Institute of Materials Chemistry, Vienna University of Technology, Getreidemarkt 9, A-1060 Vienna, Austria; 8 Institute of Life Technologies, HES-SO Valais-Wallis, Route du Rawyl 64, CP, 1950 Sion 2, Switzerland

**Keywords:** electrode, hydrogen evolution reaction (HER), magnetron sputter deposition, MoS_2_, reticulated vitreous carbon (RVC) foam, SiO_2_/Si substrate

## Abstract

Molybdenum disulphide (MoS_2_) thin films have received increasing interest as device-active layers in low-dimensional electronics and also as novel catalysts in electrochemical processes such as the hydrogen evolution reaction (HER) in electrochemical water splitting. For both types of applications, industrially scalable fabrication methods with good control over the MoS_2_ film properties are crucial. Here, we investigate scalable physical vapour deposition (PVD) of MoS_2_ films by magnetron sputtering. MoS_2_ films with thicknesses from ≈10 to ≈1000 nm were deposited on SiO_2_/Si and reticulated vitreous carbon (RVC) substrates. Samples deposited at room temperature (RT) and at 400 °C were compared. The deposited MoS_2_ was characterized by macro- and microscopic X-ray, electron beam and light scattering, scanning and spectroscopic methods as well as electrical device characterization. We find that room-temperature-deposited MoS_2_ films are amorphous, of smooth surface morphology and easily degraded upon moderate laser-induced annealing in ambient conditions. In contrast, films deposited at 400 °C are nano-crystalline, show a nano-grained surface morphology and are comparatively stable against laser-induced degradation. Interestingly, results from electrical transport measurements indicate an unexpected metallic-like conduction character of the studied PVD MoS_2_ films, independent of deposition temperature. Possible reasons for these unusual electrical properties of our PVD MoS_2_ thin films are discussed. A potential application for such conductive nanostructured MoS_2_ films could be as catalytically active electrodes in (photo-)electrocatalysis and initial electrochemical measurements suggest directions for future work on our PVD MoS_2_ films.

## Introduction

Molybdenum disulphide (MoS_2_) is a layered chemical compound comprised of covalently bonded, hexagonally coordinated S–Mo–S layers, bonded to neighbouring layers by weak van der Waals forces [1–3]. One of the unique features of MoS_2_ is the polymorphism with distinct electronic characteristics [4]. Depending on the arrangement of S atoms, several distinct symmetries may form, of which the 2H (trigonal prismatic *D*_3h_) and the metastable 1T (octahedral *O*_h_) forms are the most common [4–6]. These two phases exhibit strikingly different electronic structures, as the 2H phase is semiconducting while the 1T phase is metallic.

In the context of low-dimensional materials, recent studies demonstrated that MoS_2_ is stable in its few- and single-layer form [7–8] (similar to graphene) and has intriguing electrical and optical properties [9]. Bulk MoS_2_ is usually of 2H type and a *n*-type semiconductor with an indirect bandgap of ≈1.3 eV [10], whereas 2H MoS_2_ monolayers were found to have a direct bandgap of ≈1.8 eV [8]. Relatively high mechanical flexibility, good optical transmittance, high current on/off ratios in field effect transistor (FET) geometries and reasonably good field effect mobilities make atomically thin MoS_2_ layers a promising candidate for flexible and transparent electronics [11–13]. To exploit these beneficial properties, in electronics large area deposition of MoS_2_ films with precisely controlled layer numbers with high crystalline quality and a low defect density is typically desired.

Beyond electronic device applications, MoS_2_ is also a promising noble metal-free catalytic material for the hydrogen evolution reaction (HER) in electrochemical water splitting, which is fundamental to a hydrogen-based energy economy [14]. Density function theory showed the feasibility of MoS_2_ supported on graphite to catalyse electrochemical hydrogen evolution at a moderate overpotential of 0.1−0.2 V [15]. Triangular edge site fragments were identified as the primarily HER active sites, whereas the basal planes are relatively inactive [15–17]. In order to facilitate a large number of HER active sites in a conductive parent material, intense research efforts towards controlled deposition of MoS_2_ for HER including control over crystalline and amorphous structure, metallic 1T polymorph, vertically aligned structures, molecular mimics for MoS_2_ edge sites, doping, intercalation and hybrid formation have been undertaken (e.g., [18] and references therein). In amorphous MoS_2_ films, deposited via simple electro-polymerization procedures, the precatalysts could be MoS_3_ or MoS_2_; the active form of the catalysts was identified as amorphous MoS_2_ [19]. Narrow molybdenum disulfide nanosheets with the edge-terminated structure and a significantly expanded interlayer were synthesized through reduction and microwave heating [18]. The expanded interlayer distance with modified electronic structure is also responsible for the observed catalytic improvement, which suggests a potential way to design newly advanced molybdenum disulfide catalysts through modulating the interlayer distance. MoS_2_ films with vertically aligned layers and thereby maximally exposing edge sites were converted from e-beam evaporated, ultrathin Mo films (≈5 nm thick) by a rapid sulfurization process in a horizontal tube furnace [20]. Furthermore, a maximised electrical conductivity in the MoS_2_ is desired for HER, in order to allow efficient charge transport through the electro-catalyst layers [21].

Both electronic and electro-catalytic applications of MoS_2_ share the key pre-requisite of a scalable and controllable fabrication technique for MoS_2_. Starting from early attempts with mechanical exfoliation [11], tremendous progress on MoS_2_ growth by chemical vapour deposition (CVD) type synthesis has recently been made [20,22–26]. An alternative fabrication route with industrial scalability and potentially offering MoS_2_ deposition on a variety of substrates is physical vapour deposition (PVD) [27–28], which includes techniques such as magnetron sputter deposition, pulsed laser ablation or evaporation [3,29–30]. In this regard, PVD offers a wide processing window in terms of attainable deposition temperatures and substrates, constituent element fluxes and kinetic energies of the charged argon ions used for sputter deposition. Thus PVD potentially provides good control over the wide range of desired structures and qualities of MoS_2_ films.

Here we present morphological, structural, spectroscopic and electrical investigation of PVD MoS_2_ thin films with thicknesses in the range of ≈10 to ≈1000 nm which were deposited by magnetron sputter deposition onto SiO_2_-coated silicon (Si) wafers and reticulated vitreous carbon (RVC) electrodes for water electrolysis. Surface morphology, structure, chemical composition, stability and electrical properties of MoS_2_ thin films deposited at room temperature (RT) and 400 °C have been studied. Finally, electrochemical HER measurements and testing of MoS_2_ coated RVC electrodes and conclusions about directions for future research towards optimisation of PVD MoS_2_ films for electrocatalytic applications are presented.

## Results and Discussion

### Surface morphology, structure and chemical composition of MoS_2_ thin films deposited at RT or 400 °C

**Figure 1:**
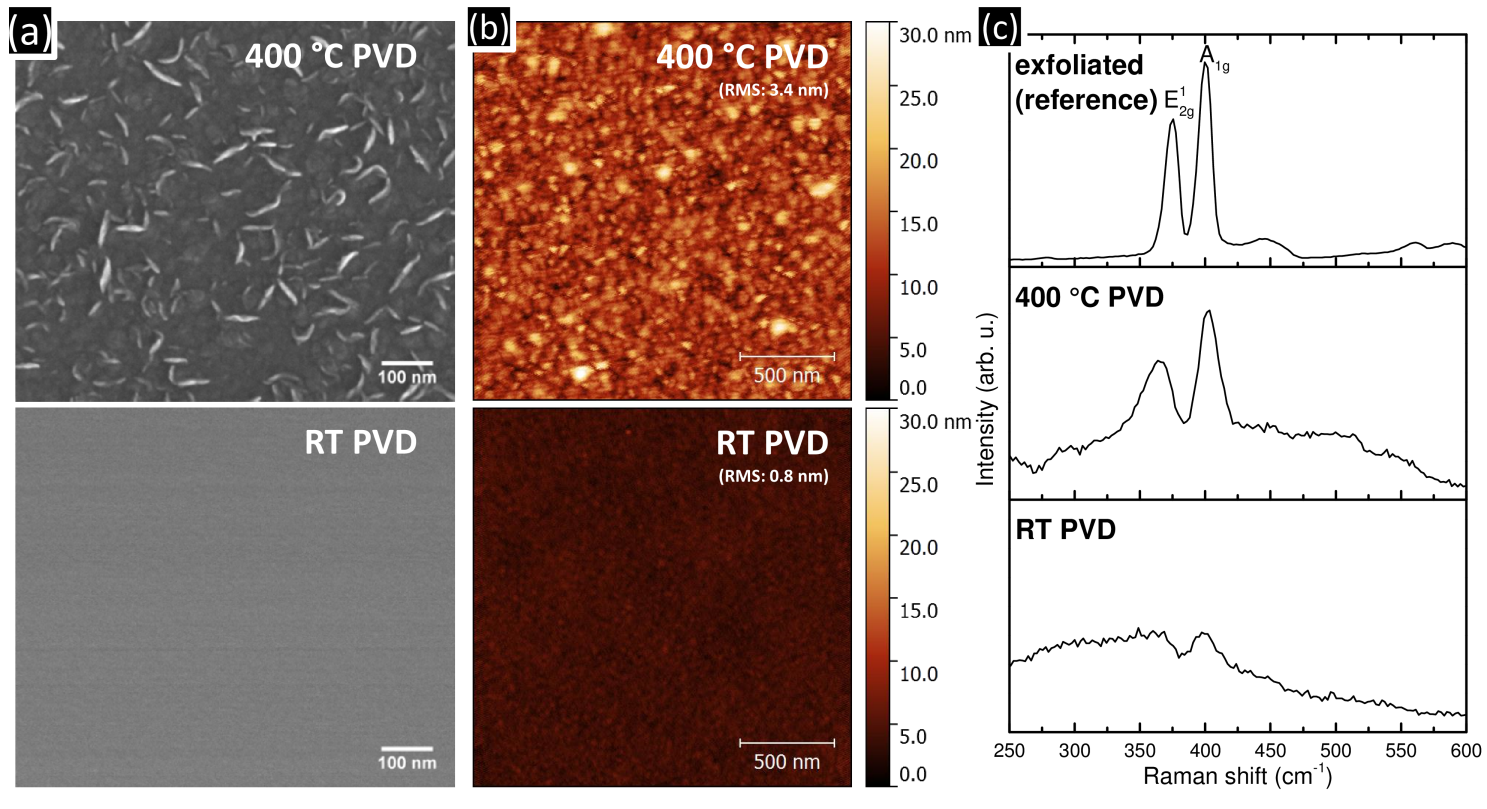
(a) SEM and (b) AFM images of MoS_2_ thin films (≈100 nm) deposited at RT and 400 °C. (c) Ex situ Raman spectra of MoS_2_ films deposited at RT (bottom, ≈1000 nm) and 400 °C (middle, ≈100 nm). The top spectrum was measured on an exfoliated MoS_2_ crystal for comparison.

The surface morphologies of our MoS_2_ thin films deposited at RT or 400 °C are displayed in Figure 1a and b. Films deposited at RT appear generally smooth, homogeneous and without topography contrast in the secondary electron scanning electron microscopy (SEM) image. This observation is confirmed by atomic force microscopy (AFM) images and a low AFM-derived root-mean-squared (RMS) surface roughness of ≈0.8 nm. No voids are detected in the films, indicating a compact morphology. At 400 °C deposition temperature the film surface appears structured in nanometer-sized grains, as visible both in the SEM and AFM images. Correspondingly, the granular surface exhibits a much higher RMS surface roughness of ≈3.4 nm. The formation of smooth MoS_2_ films during RT PVD compared to a nano-grained rougher surface for 400 °C PVD films is in excellent agreement with previous literature [31].

The corresponding Raman spectra of the films from RT and 400 °C depositions (Figure 1c), acquired ex situ on the films surfaces, show only weak and broad bands for RT deposited films and two clear bands for films deposited at 400 °C. These two bands can be assigned to the *E*^1^_2g_ and the *A*_1g_ modes, respectively, which are the typical fingerprints of MoS_2_ [32], as also seen in the reference spectrum of an exfoliated reference MoS_2_ crystal (top spectrum in Figure 1c). The increased full width at half maximum (FWHM) of the *E*^1^_2g_ and the *A*_1g_ modes for the 400 °C deposited film compared to the exfoliated reference suggest a nanocrystalline structure of the film deposited at 400 °C [33]. The even weaker Raman fingerprint in the RT deposited films suggests that the RT films are of comparably low structural order, i.e., amorphous [34].

**Figure 2:**
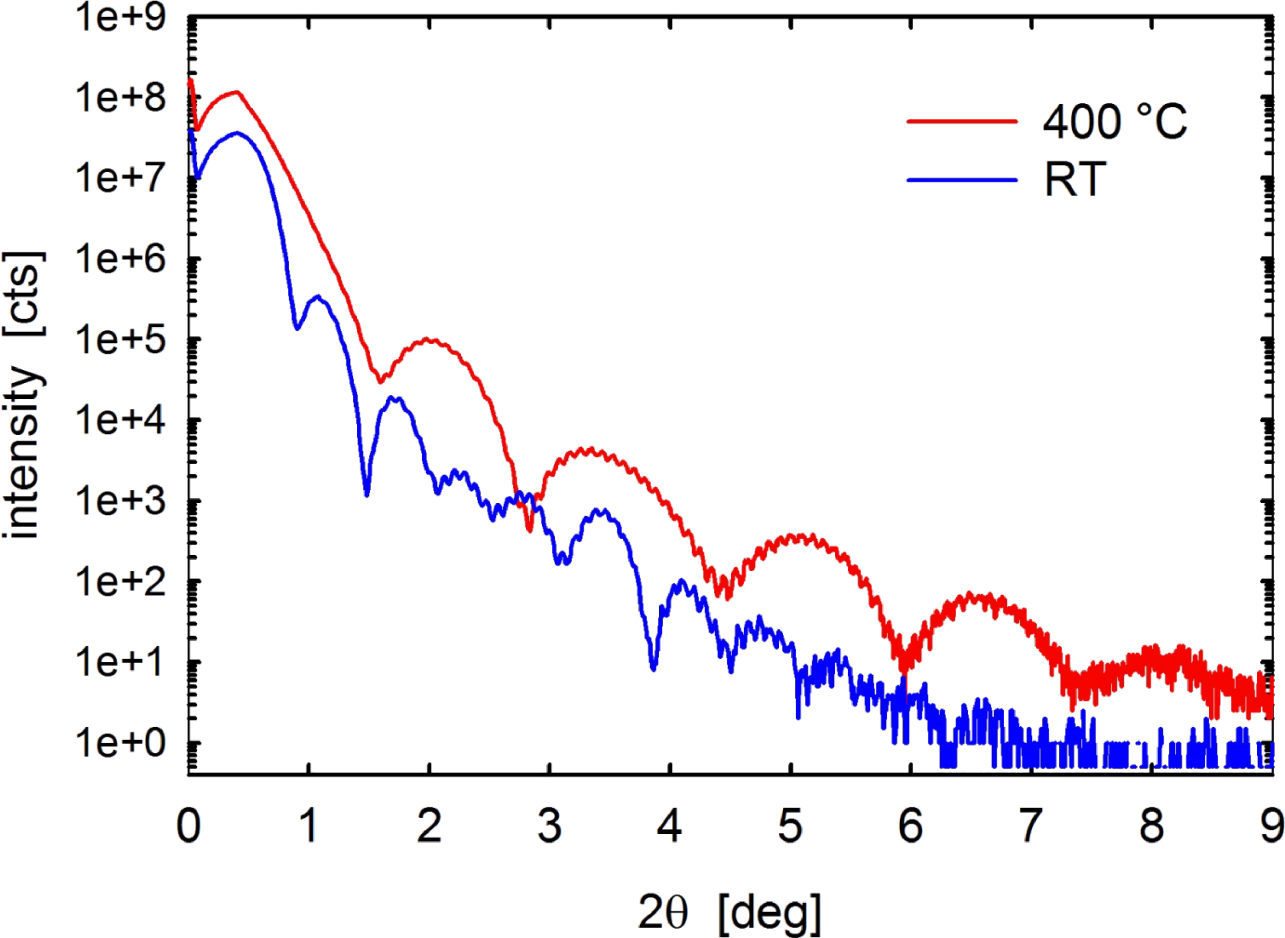
XRR pattern of MoS_2_ thin films (≈10 nm) deposited at RT and 400 °C. X-ray wavelength λ = 0.1518 nm.

X-ray reflectivity (XRR) measurements provide further insights into the properties of our MoS_2_ films. Figure 2 and Table 1 give XRR-patterns and resulting data on film thickness, surface roughness and mass density of the two films (≈10 nm) deposited at RT and 400 °C, respectively. The thickness estimates (*d*_MoS2_) of nominally 10 nm thick films yielded 13 nm for RT and 6 nm for 400 °C films. The thickness of the underlying thermal oxide SiO_2_ layer on Si (*d*_SiO2_) is about 95 nm for both. Film and substrate thickness calibrations were also corroborated by transmission electron microscopy (TEM) of focussed-ion-beam (FIB) prepared cross-sections of the PVD MoS_2_ films. According to the fit of the experimental XRR curves (not shown here) the surface roughness (σ_surf_) is ≈1.2 nm for the RT film and ≈0.6 nm for films deposited at 400 °C. While the σ_surf_ of the RT films is consistent with the AFM results (0.8 nm), the clearly lower value for 400 °C films significantly differs from the AFM derived data (3.4 nm). This may arise from the different MoS_2_ film thicknesses in the AFM (≈100 nm) compared to the XRR (≈10 nm) measurements. For the thinner films measured in XRR the size of the MoS_2_ nano-grains may still be limited by the overall film thickness, resulting in a smoother film at smaller thickness, i.e., increasing roughening of film surface occurs with increasing deposition time due to nanocrystal growth, in agreement with previous literature reports [29,35]. The interface roughness (σ_interface_) between the MoS_2_ and the SiO_2_ support is around 0.3 to 0.4 nm at both deposition conditions. This low interface roughness indicates that the SiO_2_ substrate surface remains intact during ion plasma pre-treatment and the magnetron sputter deposition process, despite the high kinetic energies of the ions in the range of several hundred eV.

**Table 1:** Thickness, roughness and density of MoS_2_ films deposited at RT and 400 °C on thermally oxidized Si/SiO_2_ wafer, determined from fitting of the XRR patterns.

Deposition temperature (°C)	RT	400

*d*_MoS2_^a^	13 ± 0.2	6 ± 0.2
*d*_SiO2_^b^	96 ± 1	95 ± 1
σ_surf_^c^	≈1.2	≈0.6
σ_interface_^d^	≈0.4	≈0.3
ρ_MoS2_^e^	≈4.3	≈3–3.9

^a^MoS_2_ layer thickness (nm); ^b^SiO_2_ layer thickness (nm); ^c^surface roughness (nm); ^d^interface roughness (nm); ^e^mass density of MoS_2_ layer (g·cm^−3^).

**Figure 3:**
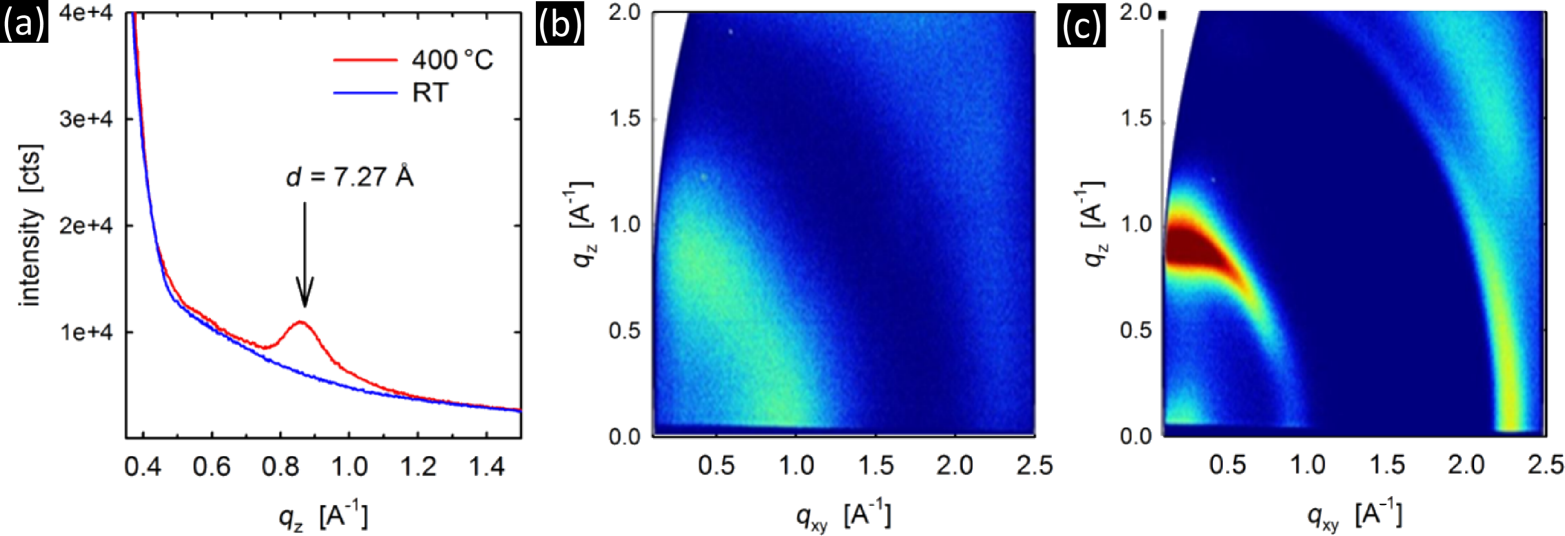
(a) Specular XRD patterns of MoS_2_ films (≈10 nm) deposited at RT and 400 °C. (b,c) GIXD images of MoS_2_ films (≈100 nm) deposited at (b) RT and (c) at 400 °C.

Fitting of the experimental data also revealed smaller electron and transferred mass densities in comparison to the known crystal structure of MoS_2_. The mass density (ρ_MoS2_) of the MoS_2_ layer was estimated to ≈4.3 and ≈3–3.9 g·cm^−3^ for films deposited at RT and 400 °C, respectively. We note that these density values are significantly smaller than the reported crystalline density of reference bulk MoS_2_ of 4.99–5.06 g·cm^−3^ [5–6,36]. The smaller mass densities can be explained by the structural imperfectness of the MoS_2_ layer, which are clearly shown by a shift in the peak position of the 00L peaks in specular X-ray diffraction (XRD) as well as broad diffraction features in grazing incidence X-ray diffraction (GIXD) experiments (Figure 3). However, the process of layer preparation is well controlled, which is reflected by the highly smooth surfaces.

The RT deposited films show no diffraction maxima, indicating an amorphous structure. This is in good agreement with the very faint Raman features for the RT film in Figure 1c as well as relatively low mass density of the RT MoS_2_ PVD films compared to bulk reference values, as estimated from XRR (Table 1). At 400 °C deposition temperature a Bragg peak around *q*_z_ of 0.9 Å^−1^ suggests a crystalline state and out-of-plane order of MoS_2_ layers with an interplanar distance of 7.27 Å. This interlayer distance is somewhat expanded compared to reported *d*-spacing of bulk reference MoS_2_ crystals of 6.1 Å [5–6] and consistent with the lower mass density determined by XRR (Table 1). From the XRD peak width of the Bragg peak at *q*_z_ of 0.9 Å^−1^ in Figure 3a a vertical crystal size of ≈8 nm can be estimated, which is in the range of the film thickness (≈10 nm), suggesting that single crystalline domains extend through the entire film thickness.

The amorphous structure of the RT films is confirmed by the almost featureless GIXD image for the thicker 100 nm films (Figure 3b). For the 400 °C deposited 100 nm films the out-of-plane features in Figure 3c at *q*_z_ of 0.9 Å^−1^ again indicate an increased interplanar *d*-spacing of 7.0 Å compared to the bulk crystalline material. The feature at *q*_xy_ 2.3 Å^−1^ (*d* = 2.73 Å) is consistent with the expected regular packing of Mo atoms within the MoS_2_ planes [5–6]. These results thus reveal an expanded stacking distance of the MoS_2_ layers in the 400 °C deposited films, whereas the in-plane structure is consistent with reference MoS_2_. The specular diffraction peak (*d* = 7.27 Å) was investigated by θ/2θ scans at different ψ angles, yielding a mosaicity of the crystallites of 8°.

The formation of amorphous MoS_2_ by sputter deposition onto substrates at RT compared to formation of nanocrystalline MoS_2_ with a certain degree of texture at elevated substrate temperatures (here, 400 °C) is in good agreement with previous reports on MoS_2_ PVD [31]. The reasons for the structural differences of our MoS_2_ thin films compared to bulk MoS_2_ reference crystals could be related to the sputter deposition process and the substrate temperature. The applied sputter deposition process employs a MoS_2_ target sputtered by argon ions. Ar (atomic number 18) preferably sputters light sulfur atoms (atomic number 16) from the target. Heavier molybdenum atoms (atomic number 42) are harder to sputter. Thus sputter and back-sputter process may result in non-stoichiometric, sulfur rich composition in our PVD films. In keeping with this, energy dispersive X-ray spectroscopy (EDX) analysis of the films in general gave slightly S-enriched Mo/S ratios of 1:2.1. Furthermore, also argon or residual oxygen might have been incorporated into the films [37–38]. This suggests that possibly S atoms (and/or other ad-atoms such as Ar, or residual co-sputtered contaminants such as metals or oxygen from the PVD system) may have been placed in between the MoS_2_ layers, resulting in the observed increased interlayer spacing and reduced mass density, whereas the in-plane ordering remains unaffected.

### Stability of MoS_2_ thin films deposited at RT and 400 °C

**Figure 4:**
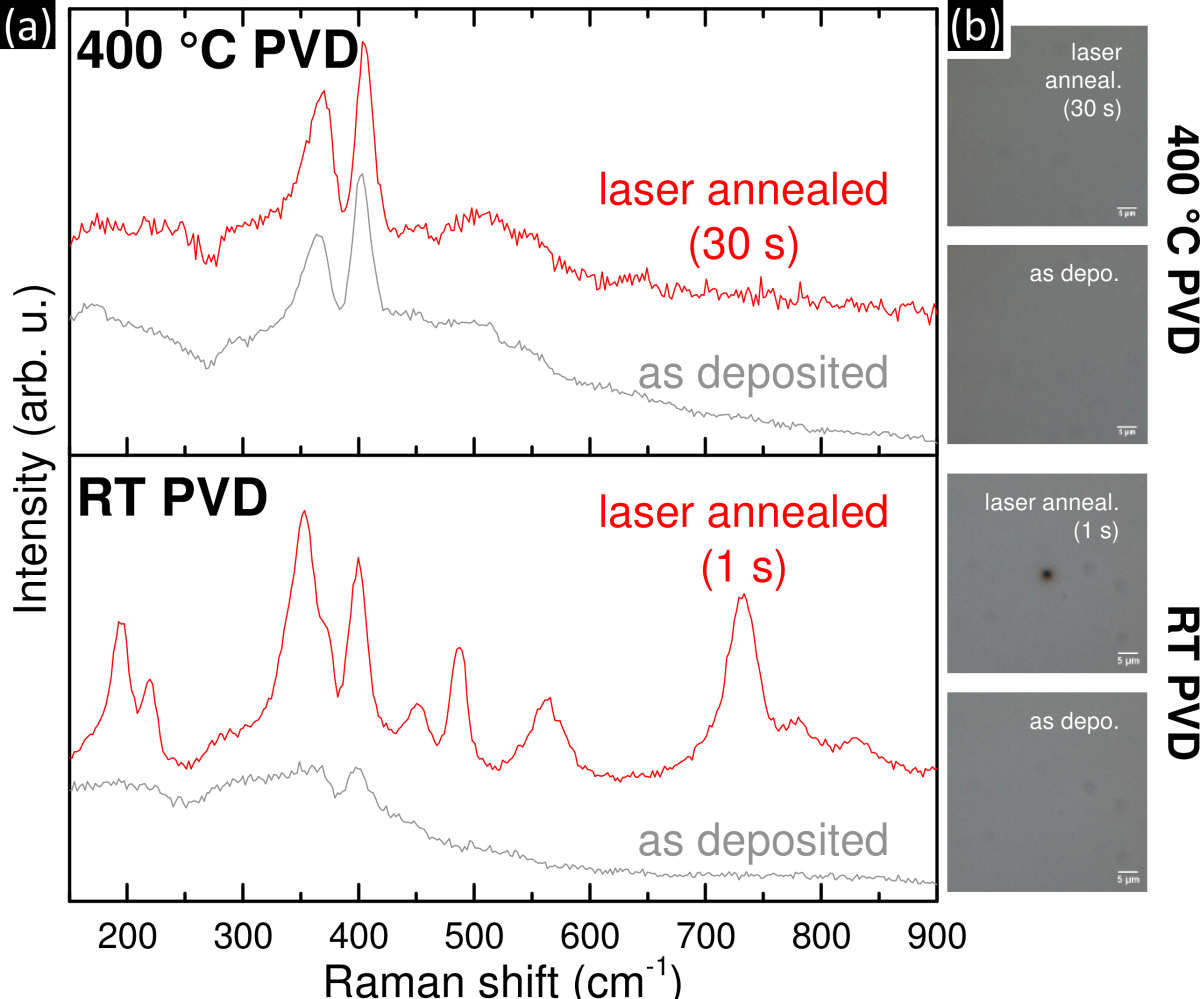
(a) In situ laser annealing Raman spectra of MoS_2_ thin films, deposited at RT (bottom panel) and 400 °C (top panel). The Raman spectra of the as deposited films (grey) were taken using a low laser power of 0.75 µW which leaves all tested MoS_2_ films unaffected. In situ laser annealing was undertaken by exposure to 3.5 mW laser irradiation for varying times in ambient atmosphere, followed by another low power 0.75 µW measurement after the intense laser anneal (red). (b) Reflected light microscopic images of the film surfaces as deposited and after laser annealing at 3.5 mW, corresponding to (a).

In view of practical applications the stability of the sputter deposited MoS_2_ thin films is of importance. Possible degradation scenarios include exposure to visible light radiation or heating to elevated temperatures in ambient atmosphere [30]. In order to study the stability of our PVD MoS_2_ films, we employed in situ laser annealing [39–40] in ambient air coupled with Raman spectroscopic investigations (Figure 4). We employed a low laser power (0.75 µW) to non-destructively probe the MoS_2_ films, and a higher laser power (3.5 mW) to test the stability of the films upon increased energy input. As described in the previous section, the spectrum and the reflected light microscopic image (Figure 4a and b bottom) of the as deposited RT film before exposed to the higher power 3.5 mW laser show broad and weak bands and a smooth surface, respectively. After 1 second exposition to the laser at 3.5 mW at a spot diameter of about 2 µm the film has been permanently modified, indicated by the development of intense and persistent new Raman bands (red spectrum in Figure 4a) as well as the appearance of a dark spot in the reflected light microscopic image (Figure 4b). The spectrum arising upon the 3.5 mW laser irradiation of the amorphous RT PVD MoS_2_ film is tentatively assigned to Mo-oxides or -hydroxides [41–42]. This suggests that through the energy input from the 3.5 mW laser the amorphous MoS_2_ disintegrates and the released Mo species are oxidized by water/oxygen in the surrounding ambient atmosphere. The exact decomposition products and pathways, e.g., release of gaseous sulphur compounds etc., are not detectable under our measurement conditions.

In contrast, the Raman spectra of films deposited at 400 °C change comparatively less when irradiated by the 3.5 mW laser for 30 seconds (Figure 4a and b top; note that this 30 seconds exposure is 30-times longer than the 1 second exposure of the amorphous films). Positions and intensity ratios of the two *E*^1^_2g_ and *A*_1g_ modes remain largely unchanged for the 400 °C film upon 3.5 mW irradiation, with only some increase in the *E*^1^_2g_ FWHM. Additionally, no surface damage is visible in the corresponding microscopic image. This suggests that the 400 °C deposited nanocrystalline MoS_2_ remains comparatively stable under these in situ annealing conditions. Our in situ laser annealing experiments therefore show that the amorphous MoS_2_ films deposited at RT are very unstable and easily oxidized upon increased energy input in ambient air whereas nanocrystalline films deposited at 400 °C remain comparatively stable in ambient conditions when exposed to visible light radiation and local temperature increase.

### Electrical properties of MoS_2_ thin films

**Figure 5:**
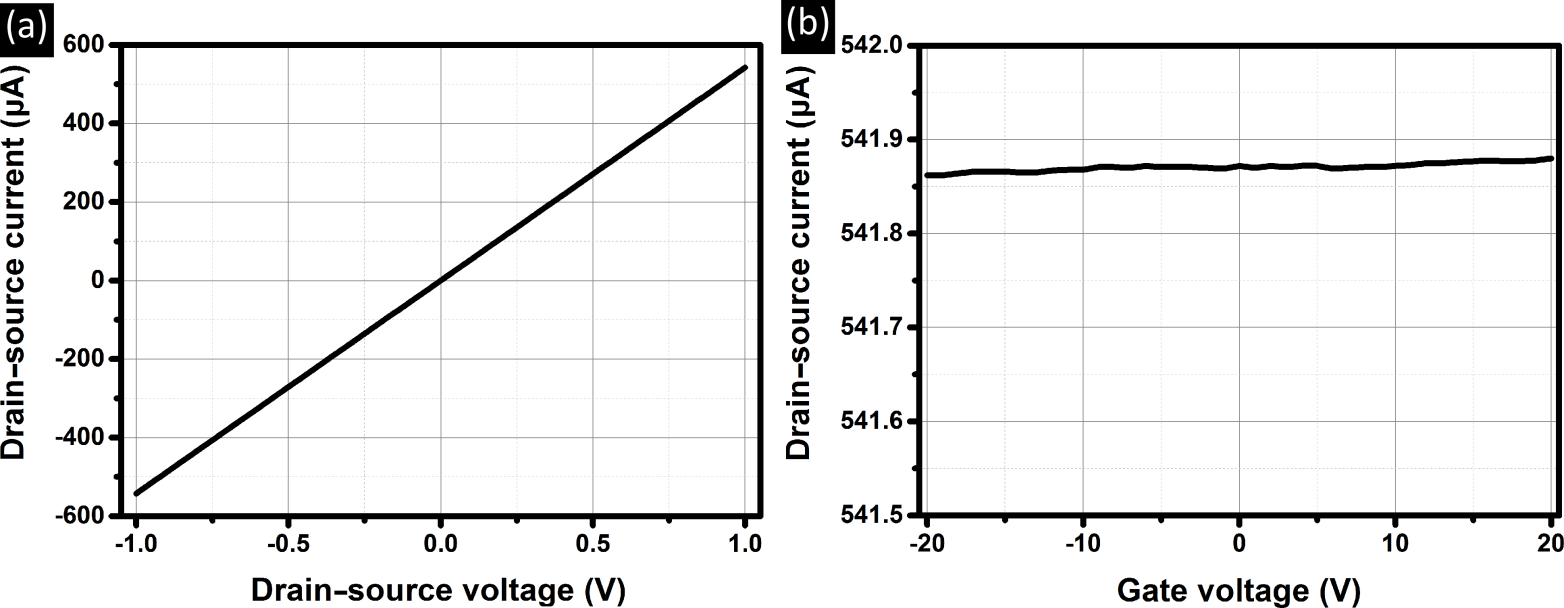
Drain–source currents measured versus the (a) drain–source and the (b) gate-voltage for a 10 nm MoS_2_ film deposited at RT. Data were obtained by utilizing the stripe contacts.

In order to investigate the electrical properties of our PVD MoS_2_ films on the SiO_2_-covered Si substrates two types of FET devices with rectangular and circular source and drain contacts were fabricated by means of optical lithography and standard contact deposition routes. As the global back gate to the FET devices the highly doped Si wafer under the 90 nm SiO_2_ film, onto which the MoS_2_ had been deposited, was employed. Electrical transport characteristics have been tested by measuring the drain-source current as a function of the gate and drain-source voltages at RT. The data for one representative 10 nm thin film deposited is shown in Figure 5. The gate leakage current in all the measurements was below 20 pA range. All electrical measurements, independent from film thickness, deposition temperature and device geometry show a linear dependence of the current on the drain–source voltage with reasonably low sheet resistances of as low as ≈25 kΩ/sq for the RT and ≈150 kΩ/sq for the 400 °C films, respectively. Surprisingly, we find negligible sensitivity of the drain–source current to the applied gate voltage for all measured films, when sweeping from −20 V to +20 V. The lack of any response to the gate voltage is inconsistent with the expected semiconducting behaviour of MoS_2_. The relatively low sheet resistance values measured here are inconsistent with the expected off-state of semiconducting MoS_2_ films [43–44]. Instead, the lack of gate-bias dependence and the reasonably high conductivity independent of gate voltage, suggests metallic-like conductivity of our PVD MoS_2_ independent of the deposition conditions. While we do find some variation in electrical properties across samples (with some devices showing higher resistances, which might however be related to non-continuous film regions under the contacts), in all our measurements we find no response to the applied gate bias. Since bulk MoS_2_ in its most stable 2H form is a semiconductor [8] the observation of such metallic-like conductivity in all our investigated PVD films was somewhat unexpected. Further work, including measurements of the temperature dependence of the film conductivities, will be necessary to confirm the exact nature of the conduction mechanisms in our PVD MoS_2_ films [45].

Possible reasons for the unexpected conduction behaviour could be related to both chemical composition and structure and will be discussed in the following. First, Mo/S stoichiometry variations in the MoS_2_ film could contribute to unexpected variations in electrical transport. For instance, for transistor devices based on CVD MoS_2_ monolayers an increase in on–off-ratios and field-effect mobility with decreasing S-content was observed [46]. Another report indicated that for highly crystalline thin films of MoS_2_, prepared by pulsed laser deposition, p-type transport (instead of the expected n-type) was observed which was attributed to excess S content induced doping [35]. Further, intrinsic defects in multilayer MoS_2_ were previously shown to dominate metal/MoS_2_ contact resistance, resulting in both n-type and p-type conduction and shifts of the Fermi level by 1 eV over tens of nanometers in spatial resolution [47]. Importantly, these variations in doping were described to be defect chemistry related and independent of contact metal. Combined this shows that variations in Mo/S stoichiometry can have severe effects on electrical transport properties of MoS_2_ films. As described above, our PVD MoS_2_ films here are generally enriched in S, where we hypothesise that this S-enrichment may (partly) cause the unusual metallic-like character. Second, besides variations in the Mo/S stoichiometry, additional add-atom species which could be incorporated during the PVD process could result in doping effects in the MoS_2_ films. Intercalation [21,48], substitution [49–50] or adsorption [51] of add-atoms in/on MoS_2_ are known to affect the electrical transport properties of MoS_2_ in a variety of ways, including change from n-type to p-type behaviour, changes in carrier numbers or changes in local structure from 2H (semiconducting) to 1T (metallic). Previous work for instance reported significant oxygen and carbon incorporation during typical PVD conditions, where substitutional doping of MoS_2_ with oxygen recently was shown to drastically alter its electronic structure [37–38,50]. Also key effects of, e.g., Nb or Na contamination on electronic properties was previously reported [29,49]. Given that the films presented in this study have been sputtered from a MoS_2_ target with a purity of 99.5 wt %, which contains 0.03 wt % SiO_2_, 0.02 wt % MoO_3_, 0.01 wt % copper oxide (CuO), 0.019 wt % iron (Fe) and up to 0.20 wt % not specified compounds, in a metallic sputter chamber under medium pressure Ar conditions several sources of contaminational add-elements could persist, such as chemical residues in target, unintentional co-sputtering from the chamber or incorporation of Ar gas or other gaseous residues etc. While we currently do not have sensitive enough measurements on our films to confirm trace contaminations, the increased interlayer distance in our 400 °C films observed in XRD could suggest that additional add-elements may (partly) be intercalated between individual MoS_2_ layers and thus alter electronic transport properties of the PVD films. Third, and partly linked with add-atom incorporation or intercalation, also local variations in the structure of MoS_2_ layers could affect conduction: An expanded interlayer distance of 9.4 Å from molecular intercalation was previously shown to modify the electronic structure of layered MoS_2_ and to improve catalytic HER performance for hydrogen production [18]. Intercalation and adsorption of species was also shown to lead to metallic 1T phase regions in semiconducting 2H MoS_2_ [48]. If a large enough fraction of 1T structure is formed in a semiconducting 2H film this could lead to a metallic percolation network, rendering the films overall metallic. Local 1T and 2H phase regions are not straightforward to differentiate by the methods thus far employed in this study, but further dedicated characterisation work could provide conclusive insights into such possible structural modifications.

### Electrochemical HER measurements and testing of MoS_2_ coated electrodes

**Figure 6:**
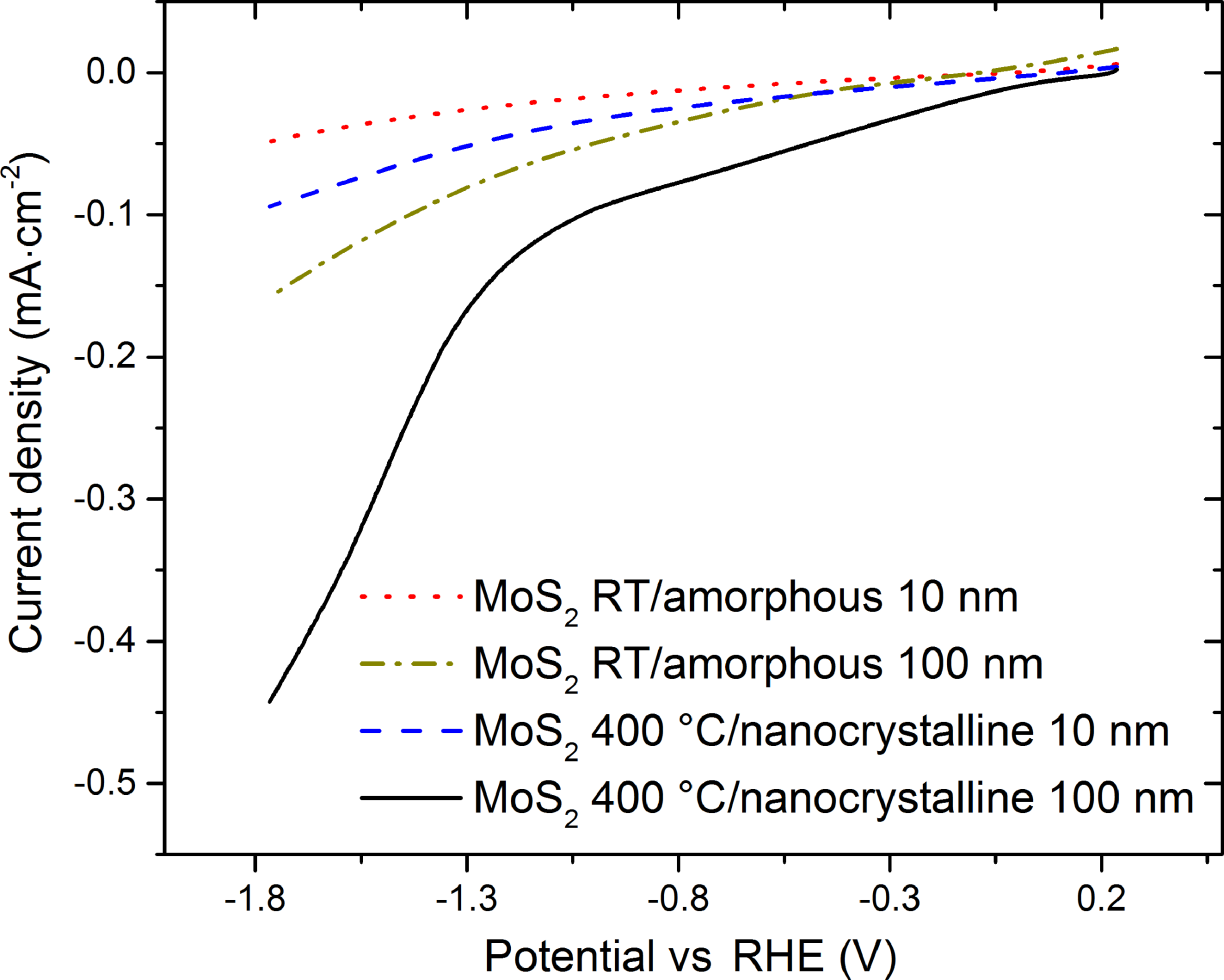
Electrochemical HER measurements on our PVD MoS_2_ films directly on SiO_2_ covered Si substrates.

Finally, we present initial electrochemical measurement with our PVD MoS_2_ films. In a first set of experiments HER measurements with our as deposited PVD MoS_2_ films directly on SiO_2_ covered Si substrates were performed. The results confirm that sputter deposition techniques are suitable for the fabrication of electrocatalytically active films. Figure 6 shows that for these HER measurements with PVD MoS_2_ on SiO_2_/Si the absolute values of required overpotentials for HER onset and the obtained current densities for this device geometry are poor compared to state-of-the-art MoS_2_ electrodes [14]. We note however that in our device geometry the MoS_2_ films are deposited directly on an electrically insulating SiO_2_ substrate which during the HER testing requires the entire current in our devices to flow along the only reasonably conductive thin MoS_2_ film over a cm length scale. In contrast, in many optimized device geometries the catalytically active MoS_2_ films are deposited onto highly conductive substrates, such as glassy carbon [18,20], where charge transport is then facilitated by current flowing only across the thin MoS_2_ film on a nm length scale into the highly conductive substrate and then along this highly conductive substrate, resulting in overall much reduced electrode resistances and thus a lower resistive potential drop along the MoS_2_ film. Nevertheless, the HER measurements of our PVD MoS_2_ on the SiO_2_/Si in Figure 6 already give some insights into the respective relative merits of our deposition conditions. Two trends are discernible: First, obtained current densities increase with increasing film thickness. This corroborates that the device performance is here limited by the resistance of the MoS_2_ film. (Kinetical limitation due to increased accessible surface area can be excluded because of the non-porous, compact film structure demonstrated in AFM and SEM surface images (Figure 1) and TEM cross sections (not shown here).) Second, the nanocrystalline MoS_2_ deposited at 400 °C shows a significantly better performance compared to the amorphous MoS_2_ from RT deposition (for both tested 10 nm and 100 nm film thicknesses). This suggests that the higher degree of crystallinity combined with the larger specific surface area from the nano-grained surface morphology in our 400 °C PVD MoS_2_ films are advantageous towards better HER electrochemical performance.

**Figure 7:**
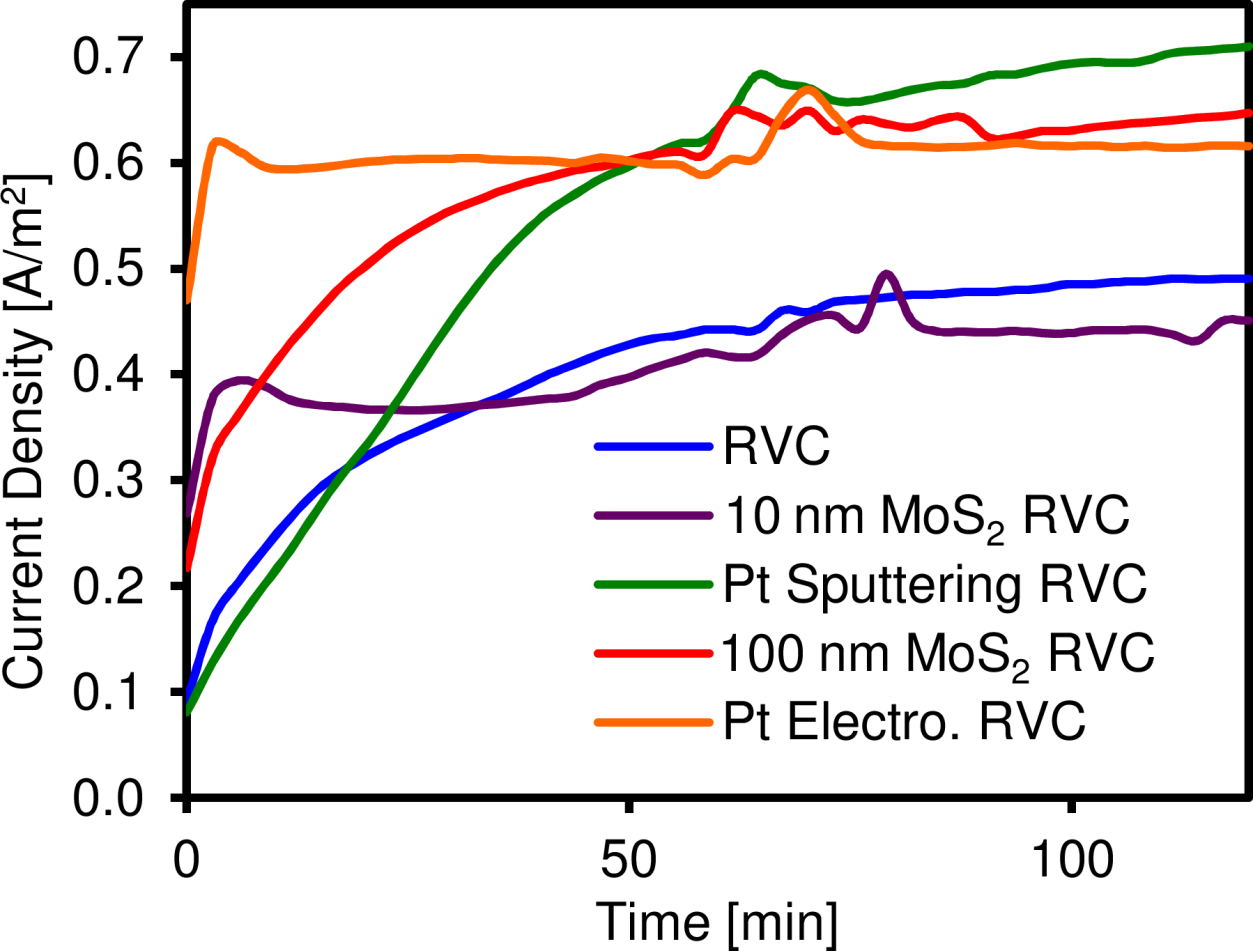
Current generated per area of the uncoated and coated electrodes over time. The currents have been normalized as a function of the RVC area of 21 cm^2^ with a total accessible surface area of 249.6 cm^2^.

These assertions are further explored by a second set of electrochemical experiments where reticulated vitreous carbon (RVC) foam electrodes coated with our PVD MoS_2_ were tested for water electrolysis. RVC foam electrodes are a commercial electrode type for bulk electrolysis applications. The advantage of the RVC electrodes used for the experiments is the high available surface area of 249.6 cm^2^ compared to its volume of 3.84 cm^3^ [52]. For electrochemical water splitting RVC foams coated with Pt are often investigated [53]. The particular electrochemical two-terminal setup employed here is aimed at realization of microbial electrolysis cells/sustainable base production in which the MoS_2_- or Pt-coated electrodes would be used on the cathodic side [54]. Here, we compared 400 °C deposited PVD MoS_2_ coatings of 10 and 100 nm thickness with two 50 and 25 nm thick reference Pt coatings prepared by PVD and electroplating, respectively. We use the MoS_2_-coated (or, for reference, Pt-coated) RVC foam electrodes on the cathodic side and a bare RVC foam electrode on the anodic side in the electrochemical two-terminal setup. The thus obtained electrode performance values are relative to the particular two-terminal setup [54] and electrode geometry but allow relative comparisons of the tested electrode materials. Figure 7 shows the current density resulting from a positive potential applied to the entire cell (anode + cathode) of 1.50 V. Both platinum coated electrode (PVD and electroplated) and the 400 °C PVD 100 nm MoS_2_ coated RVC had a similar performance, superior to the blank RVC and the 400 °C PVD 10 nm MoS_2_ coated RVC electrode.

**Figure 8:**
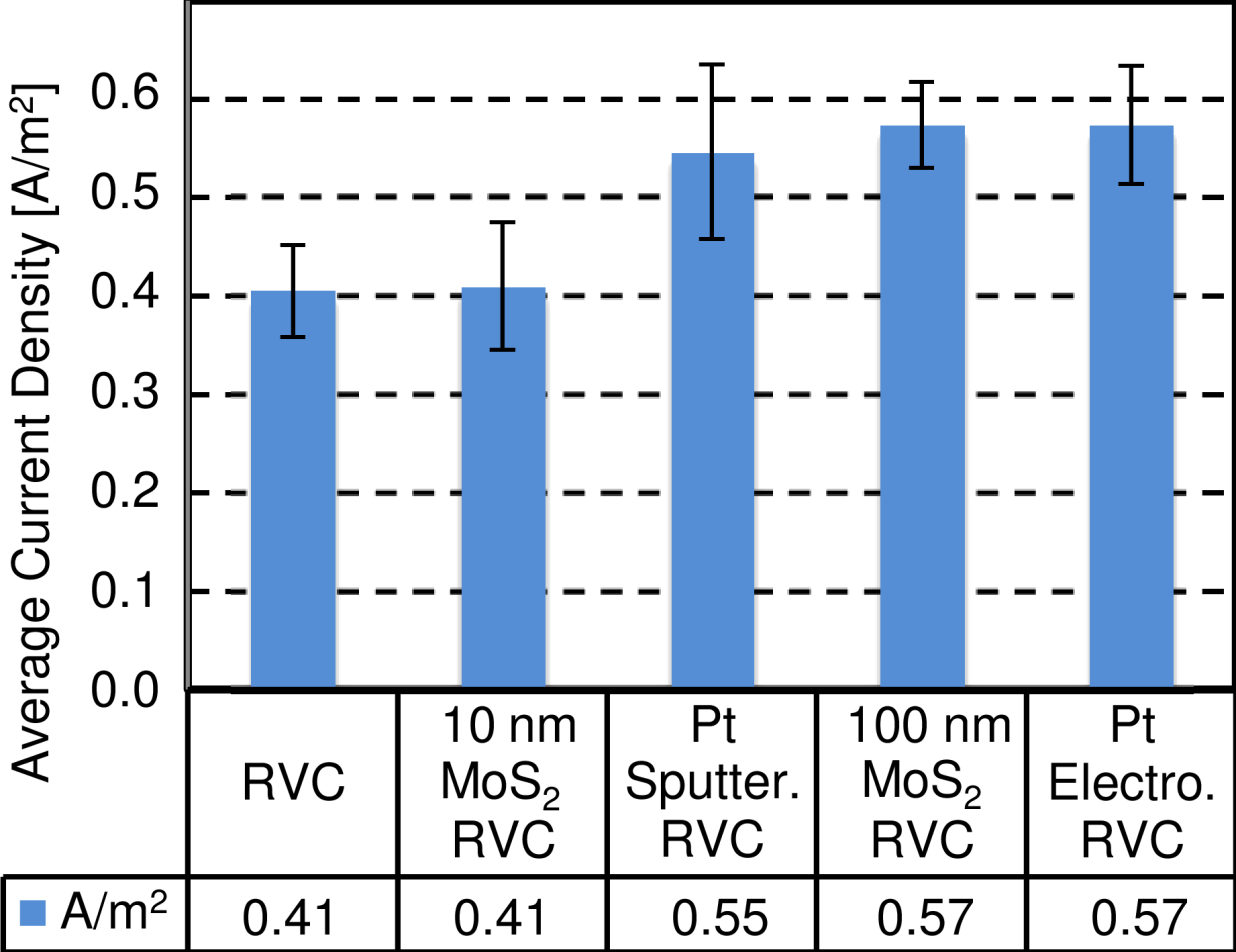
Average current density generated per uncoated and coated electrodes during the 2 h experiments. The currents have been normalized as a function of the RVC area of 21 cm^2^.

The same findings are observable when calculating the average current density of the system (Figure 8). Indeed, as previously mentioned, the current (normalized as a function of the RVC area) generated by the 10 nm MoS_2_ coating is identical to the blank (RVC without coating) (0.41 A/m^2^). On the contrary, the 100 nm MoS_2_ coated electrode and both platinum RVC are 35 to 40% more efficient regarding the generated current density (0.55–0.57 A/m^2^). The current produced by the MoS_2_ coatings depends on their thickness, whereas almost identical values have been obtained for the two 25 and 50 nm thick platinum coatings.

**Figure 9:**
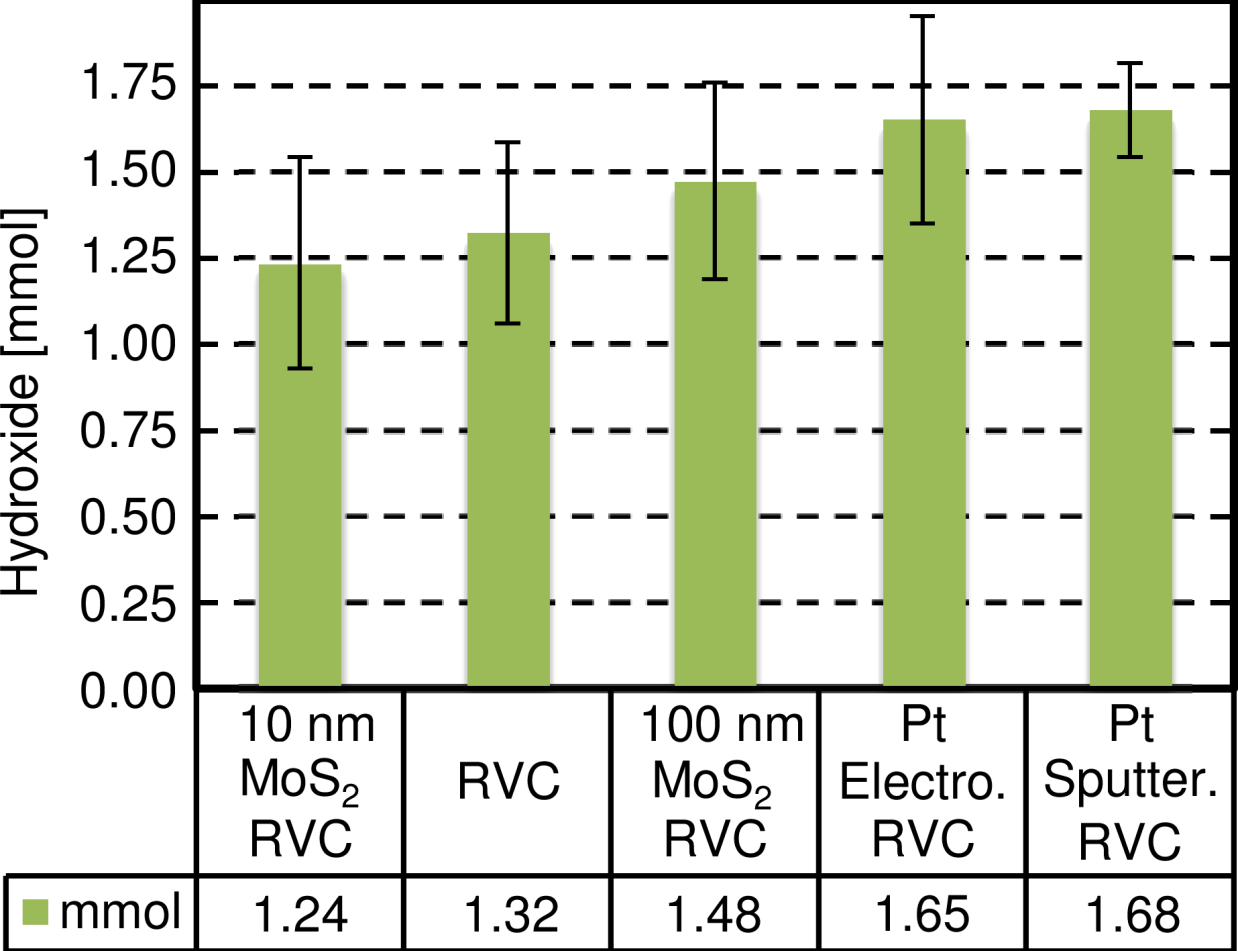
Average hydroxide (OH–) moles produced during the 2 h experiments.

The RVC coatings were also compared in terms of mole of hydroxide produced. The quantity of hydroxide produced during the entire time of the process was determined by testing the pH of the catholyte at the end of the reaction (after 2 h). Figure 9 displays these measurements.

Although the results are not as significant as for the average generated current density the same trend can be observed: The amount of produced hydroxide moles with the 10 nm MoS_2_ coating (1.24 mmol) is similar to the uncoated RVC (1.32 mmol). The 100 nm MoS_2_ coating enhanced the hydroxide production to 1.48 mmol (≈+12%). The best results were obtained with platinum coated electrodes (>1.65 mmol, >+25%).

Platinum coatings generated a similar current but produced a higher number of hydroxide moles compared to 100 nm MoS_2_ coated electrodes, showing the better catalytic power of platinum regarding base production (water electrolysis). However, as it has been demonstrated by both HER experiments on SiO_2_ and the RVC study, it can be expected that optimized MoS_2_ coatings can compete with the performance of platinum at a fraction of its costs.

## Conclusion

In summary, we have explored the parameter space of PVD of MoS_2_ thin films. We find that RT-deposited MoS_2_ films are amorphous, have a smooth surface and readily degrade during laser-induced annealing in ambient atmosphere. In contrast, films deposited at 400 °C are nano-crystalline, show a nano-grained surface morphology and are comparably stable against degradation. All our studied PVD MoS_2_ films exhibit a non-stoichiometric, S-rich composition and reduced mass density. The 400 °C deposited films exhibit increased interlayer spacing and textured microstructure. Importantly both RT and 400 °C deposited films appear to have a metallic-like conduction character, which is unexpected for MoS_2_ films that are usually semiconducting. It is likely that a combination of Mo/S stoichiometry, add-atom contamination and local structural modifications causes the observed unusual electrical properties of our films. Further work will be necessary to confirm and identify the exact origin of the unusual metallic-like conduction. We note however that the metallic-like conductivity of our sputter deposited MoS_2_ films in conjunction with their nanocrystalline structure and stability at increased temperatures (in particular for the 400 °C films) may make them interesting for possible applications as catalyst films in the field of (photo-)electrochemical water splitting. Initial electrochemical measurements suggest directions for future work towards electrocatalysis applications of our PVD MoS_2_ films.

## Experimental

### Magnetron sputter deposition

MoS_2_ deposition was undertaken in a modified, industrially compatible sputtering plant (Pfeiffer Vakuum, Germany). Thin films have been sputter deposited by an unbalanced cathode from AJA (AJA International, North Scituate, MA, USA) and a 6 mm thick MoS_2_ target with 76.2 mm (3-inch) diameter (Sindlhauser Materials GmbH, Germany). Purity of the target is 99.5 wt % MoS_2_, according to the materials test certificate further compounds are SiO_2_ (0.03), MoO_3_ (0.02), CuO (0.01), Fe (0.019) and not specified elements (<0.20). As substrates silicon wafers (100) with ≈100 nm thermal SiO_2_ layer were used, which were cleaned with isopropanol and dried with nitrogen prior to deposition. The substrates were fixed on a static, floating potential substrate holding device kept at approximately ten centimetres away from the sputtering target. The substrate temperature was held at RT or 400 °C and monitored with an electrically insulated K-type thermocouple (Jumo, Fulda, Germany) installed at the backside of the heated substrate holder. After pumping to medium vacuum conditions (2 × 10^−3^ Pa), an ion plasma pre-treatment with a linear anode layer ion source (Veeco ALS 340, Fort Collins, CO, USA) was performed on the substrates. For the MoS_2_ deposition, the cathode was powered with a 10 kW DC power supply in power regulation mode. Pulse frequency of 80 kHz, pulse time 1 µs and power 150 W, power density ≈3.3 W·cm^−2^, was applied to the target, resulting in voltages around 600 V. Argon 5.0 (nominal purity >99.999%) at a pressure around 6 × 10^−2^ Pa was used as sputtering gas. Coating thickness was controlled by deposition times estimated from preliminary experiments and cross-checks via XRR measurements and TEM imaging of FIB-cut film cross-sections. The large number of experimental characterisation methods required comparison of different film thicknesses, however all give a consistent picture of the film properties.

### Atomic force microscopy (AFM) and Raman spectroscopy

A NT-MDT Ntegra Spectra coupled AFM and Raman spectrometer [55] was used for AFM imaging in tapping mode. For Raman spectroscopy an excitation wavelength of 473 nm was employed. For modification-free measurements on the MoS_2_ films a low laser power of 0.75 µW was employed while for in situ modifications to the film a higher laser power of 3.5 mW was used. The laser power was selected by neutral density filters and calibrated using a power-meter. The laser spot diameter is estimated to ≈2 µm. The in situ annealing Raman experiments were conducted in ambient atmosphere (23 °C, 30% humidity).

### X-ray reflectivity (XRR) and specular diffraction (XRD)

X-ray reflectivity (XRR) and specular X-ray diffraction was performed on a PANalytical Empyrean reflectometer using Cu Kα radiation (wavelength λ = 0.1518 nm). At the primary side a multilayer mirror was used for monochromatisation and parallelizing the beam and at the secondary side, a receiving slit, a Soller slit and a PANalytical PIXcel^3D^ detector were used.

### Grazing incidence X-ray diffraction (GIXD)

Grazing incidence X-ray diffraction (GIXD) measurements were performed at the KMC-2 beamline at BESSY II (Berlin, Germany) using X-rays with a wavelength of 1.00 Å and a 2D cross-wire detector (BRUKER). An incident angle of α_i_ = 0.13° was chosen to enhance the scattered intensities of the adsorbate. The angular scans have been transferred to scattering vector notation using *q* = 4πsin(Θ)/λ*xt*.

### Scanning electron microscopy (SEM) and energy dispersive X-ray spectroscopy (EDX) and focused ion beam (FIB) preparation and transmission electron microscopy (TEM)

Secondary electron images were collected using an Everhart–Thornley-detector mounted on a FEI Quanta 3D FEG applying electron beam settings of 15 kV accelerating voltage. In the same system cross-sectional electron transparent foils of the MoS_2_ films on the SiO_2_/Si support were fabricated using focussed ion beam (FIB) sputtering at IB settings of 30 kV accelerating voltage and successively decreasing IB currents from 65 nA to 50 pA. The 90–120 nm thick sample foils were subsequently checked for film thickness accuracy determination in a Philips CM200 TEM at 150 kV. EDX spectroscopy was performed in a Zeiss Supra 55VP SEM at 20 kV using an Oxford Instruments X-max detector and the INCA software for elemental composition quantification.

### Electrical measurements

To investigate the conductivity of the MoS_2_ films two types of field effect transistor (FET) devices with rectangular and circular contacts have been made. The drain/source contacts were defined by means of optical lithography. The Ti/Au (5/50 nm) contact pads have been fabricated by means of thermal evaporation. As the global back gate to the FET devices the highly doped Si wafer under the ≈100 nm SiO_2_ film, onto which the MoS_2_ had been deposited, was employed. Electrical transport characteristics have been tested by measuring the drain-source current as a function of the gate and drain–source voltage. The gate leakage current in all the measurements has been below 20 pA.

### Electrochemical HER measurements and RVC electrode testing for water electrolysis

Electrochemical HER tests were conducted on as deposited PVD MoS_2_ films directly on the SiO_2_ (≈100 nm) covered Si substrates (≈1 cm × ≈2 cm). The MoS_2_ films were electrically contacted on one sample edge from the film top side using carbon paste (vacuum-dried at 50 °C). This resulted in a solution immersed sample area of ≈1 cm × ≈1 cm (precise immersed area for each sample calculated after electrochemical testing using digital photographs). A three electrode electrochemical setup was employed, with Pt as the counter electrode and Ag/AgCl as the reference electrode. Ag/AgCl data was recalculated to yield potential versus reversible hydrogen electrode (RHE) values for easier comparison with the wider literature. 0.1 M Na_2_SO_4_ with pH close to neutral was used as the electrolyte.

Further electrochemical testing for water electrolysis was done using reticulated vitreous carbon (RVC) foam (ERG aerospace corporation, USA) electrodes with 100 pores per inch, specific surface area ≈65 cm^2^·cm^−3^, total projected area 21 cm^2^, volume 3.84 cm^3^ and total accessible surface area of 249.6 cm^2^ [52]. Two 50 and 25 nm thick platinum coatings, produced at HES-SO Valais-Wallis, Switzerland by PVD and electro-plating, respectively, and 10 and 100 nm thick sputtered MoS_2_ films deposited at 400 °C were applied to the RVC. For electrochemical tests a small-scale reactor with both a cathodic and anodic compartments was used [54]. The coated RVC electrodes were loaded into the cathodic compartment while a bare RVC reference electrode was loaded into the anodic chamber. A solution of 0.01 M sodium sulphate was used as anolyte. The cathodic compartment was filled with demineralized water (30 mL). It was connected to a peristaltic pump in order to provide a constant agitation (32 mL/min). When considering the complete system a positive potential of 1.50 V was applied by an external power supply. A multimeter was connected to the output of a decade box set up with a 10 Ω-resistance to measure the current generated by the system. The duration of each experiment was 120 minutes. A pH meter was used to control the hydroxide concentration at beginning (0 h), middle (1 h) and end of the process (2 h). Each experiment has been realised 3 times and the average of generated hydroxide was calculated from the pH of the obtained base.
